# Atheroprotective mechanism by which folic acid regulates monocyte subsets and function through DNA methylation

**DOI:** 10.1186/s13148-022-01248-0

**Published:** 2022-02-28

**Authors:** Yang Xiang, Bin Liang, Xiaokang Zhang, Xueping Qiu, Qianyun Deng, Li Yu, Hong Yu, Zhibing Lu, Fang Zheng

**Affiliations:** 1 Center for Gene Diagnosis, and Department of Clinical Laboratory Medicine, Zhongnan Hospital of Wuhan University, Donghu Road 169, Wuhan, 430071 China; 2 Laboratory Medicine, Guangdong Provincial People’s Hospital, Guangdong Academy of Medical Sciences, Guangzhou, China; 3 Hubei Provincial Key Laboratory of Developmentally Originated Disease, Wuhan University School of Basic Medical Sciences, Wuhan, 430071 Hubei China; 4 Institute of Myocardial Injury and Repair, Zhongnan Hospital of Wuhan University, Donghu Road 169, Wuhan, 430071 China

**Keywords:** Coronary artery disease, DNA methylation, Monocyte subsets, ARID5B, Folic acid

## Abstract

**Background:**

Recent studies have suggested that folic acid can restore abnormal DNA methylation and monocyte subset shifts caused by hyperhomocysteinemia (HHcy) and hyperlipidemia (HL). However, the exact mechanism of action is still not fully understood. In this study, we further investigated the reversal effect and underlying mechanism of folic acid on the shift in monocyte subsets induced by aberrant lipids and Hcy metabolism via DNA methylation in vitro and in vivo.

**Results:**

Our results showed that intermediate monocytes were significantly increased but had the lowest global 5-methylcytosine (5-mC) levels in coronary artery disease (CAD) patients, which might lead to a decrease in the global 5-mC levels of peripheral blood leukocytes (PBLs). We also discovered that ARID5B might mediate the increased proportion of intermediate monocytes, as this factor was related to the proportion of monocyte subsets and the expression of CCR2. The expression of ARID5B was inversely associated with the hypermethylated cg25953130 CpG site, which was induced by HL and HHcy. ARID5B could also regulate monocyte CCR2, MCP-1, and TNF-α expression, adhesion and migration, macrophage polarization, and monocyte/macrophage apoptosis, which might explain the regulatory effect of ARID5B on monocyte subset shifting. Folic acid reversed HL- and HHcy-mediated aberrant global and cg25953130 DNA methylation, reduced the proportion of intermediate monocytes, and inhibited the formation of atherosclerotic plaques.

**Conclusion:**

Folic acid plays a protective role against atherosclerosis through the regulation of DNA methylation, ARID5B expression, and monocyte subsets.

**Supplementary Information:**

The online version contains supplementary material available at 10.1186/s13148-022-01248-0.

## Introduction

Atherosclerosis (AS) is the pathological cause leading to coronary artery disease (CAD), and its risk factors include hyperhomocysteinemia (HHcy), hyperlipidemia (HL), and abnormal epigenetic mechanisms associated with gene–environment interactions [1, 2].

Monocytes play a key role in the progression of AS and present considerable heterogeneity [3]. Based on the expression of CD14, CD16, and CCR2, human monocytes can be defined as classical (CD14^++^CD16^−^CCR2^++^), intermediate (CD14^++^CD16^+^CCR2^+^), and nonclassical (CD14^+^CD16^++^CCR2^−^) subsets [4], and changes in the expression of these markers lead to shifts in monocyte subsets [5]. Similarly, mouse monocytes can be divided into classical (Ly6C^++^CD43^+^), intermediate (Ly6C^++^CD43^++^), and nonclassical (Ly6C^+^CD43^++^) subsets [6]. These subsets display distinct phenotypic and functional features during the progression of AS [7]. As the smallest subset with increased inflammatory properties and proportions, intermediate monocytes have gained attention in the field of AS [8, 9]. In addition, intermediate monocytes have been associated with the occurrence and outcome of arteriosclerotic cardiovascular disease (ASCVD) [10, 11].

Multiple AS risk factors, such as dyslipidemia and HHcy, have been associated with alterations in monocyte subsets [12, 13]. Animal studies revealed that HHcy promoted the generation of Ly6C^mid^ intermediate monocytes [14]. Zawada et al*.* [13] suggested that the DNA demethylation caused by the Hcy hydrolysate S-adenosylhomocysteine (SAH) mediates the increase in the proportion of intermediate monocytes. In addition, Hcy could induce the upregulation of CD40 through DNA hypomethylation, thereby promoting the differentiation of CD40 + inflammatory intermediate monocytes [15]. Moreover, studies have shown that aberrant lipid and homocysteine (Hcy) metabolism contributes to aberrant DNA methylation [16, 17] and abnormal expression of CD14, CD16, and CCR2 on monocytes [18, 19]. However, to date, only a few studies have reported that aberrant DNA methylation caused by HL and HHcy modulates monocyte subset shifts in CAD.

A recent study revealed that ARID5B regulates monocyte phagocytosis and the expression of typing markers in cardiovascular disease (CVD) and that the methylation of cg25953130 is inversely associated with its expression [20]. Cg25953130 is located in an enhancer region [20] in the third intron of ARID5B (chr10:63,753,550–63,753,550, GRCh37/hg19). However, whether ARID5B methylation mediates the shifts in monocyte subsets is not clear.

Folic acid is involved in regulating lipid and Hcy metabolism [21, 22], restoring abnormal DNA methylation [23], and regulating monocyte phenotype ^15^. Therefore, in the present study, we explored the protective mechanism of folic acid against AS; we hypothesized that folic acid regulates monocyte subsets and functions by affecting DNA methylation and ARID5B.

## Results

### Global 5-mC levels are negatively associated with the proportion of intermediate monocytes in CAD patients

The clinical characteristics of the enrolled subjects (180 CAD vs. 210 control) are presented in Additional file 2: Table S1. Age and gender distributions were similar among CAD patients and healthy controls (*p* > 0.05). However, CAD patients had significantly higher proportions of T2DM and hypertension; higher serum FPG, TG, and Hcy levels; and lower serum TC and HDL-C levels than healthy controls (all *p* < 0.05). In addition, CAD patients had higher monocyte counts (*p* < 0.05). We randomly selected 112 healthy controls and 110 CAD patients and examined the expression levels of ARID5B and DNMT1 and the methylation level of cg25953130. The clinical characteristics of the randomly selected subjects are presented in Additional file 2: Table S2. The significant differences were consistent with the overall sample characteristics.

**Fig. 1:**
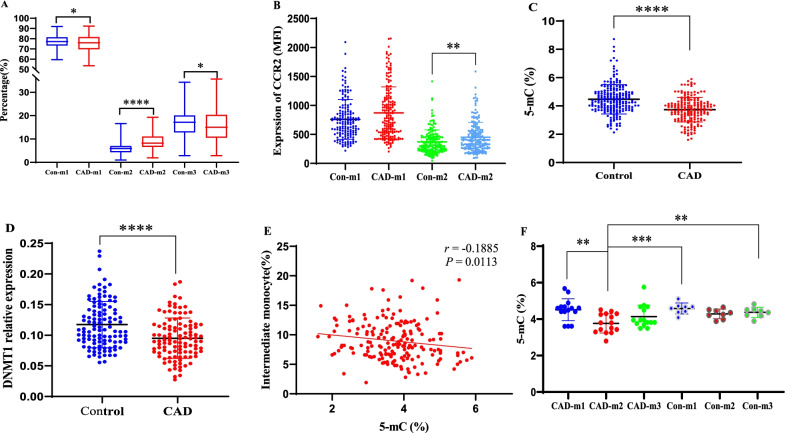
Global 5-mC levels are negatively associated with the proportions of intermediate monocytes in CAD patients. **A** The proportions of monocyte subsets in CAD patients (*n* = 180) and healthy controls (*n* = 210) (%). **B** The expression levels (MFI) of CCR2 on monocyte subsets. **C** Global 5-mC levels in PBLs in CAD patients (*n* = 180) and healthy controls (*n* = 210) (%). **D** The relative mRNA expression of DNMT1 in CAD patients (*n* = 110) and healthy controls (*n* = 112). **E** Spearman correlation between global 5-mC levels and the proportions of intermediate monocytes in CAD patients (*n* = 180). **F** Global 5-mC levels in the monocyte subsets in the CAD (*n* = 15) and control (*n* = 8) groups (%). m1, classical monocytes; m2, intermediate monocytes; m3, nonclassical monocytes; MFI, median fluorescence intensity; Con, control; CAD, coronary artery disease; 5-mC, 5-methylcytosine. *p < 0.05, **p < 0.01, ***p < 0.001, ****p < 0.0001

A shift in monocyte subsets and global DNA demethylation was observed in CAD patients. As shown in Fig. 1A, the proportion of intermediate monocytes was significantly increased in CAD patients, while the proportions of classical and nonclassical monocytes were significantly decreased (all *p* < 0.05). In addition, the expression level of CCR2 on intermediate monocytes was significantly elevated in CAD patients (*p* < 0.05), while the increase in CCR2 expression on classical monocytes was not significant (*p* = 0.0512) (Fig. 1B).

Global 5-mC levels in PBLs were significantly decreased in CAD patients (*p* < 0.05, Fig. 1C), which was consistent with our previous study [24]. Correspondingly, the expression of DNMT1 was significantly reduced in CAD patients (*p* < 0.05, Fig. 1D). In addition, global 5-mC levels in PBLs were negatively associated with the proportions of intermediate monocytes (*r* = − 0.1885, *p* = 0.0113, Fig. 1E) in CAD patients.

Our previous study indicated that the decrease in global 5-mC levels in PBLs was mainly associated with monocytes [24]. In the present study, we found a negative association between global 5-mC levels in PBLs and the proportion of intermediate monocytes. To further explore the causal relationship, we measured global 5-mC levels in monocyte subsets. In CAD patients, classical monocytes had the highest global 5-mC levels, followed by nonclassical and intermediate monocytes; in addition, the global 5-mC levels in classical and intermediate monocytes were significantly different (*p* < 0.05, Fig. 1F). However, the global 5-mC levels in monocyte subsets in the control group showed no significant differences (*p* > 0.05, Fig. 1F).

### ARID5B is associated with the proportion of monocyte subsets and CCR2 expression

**Fig. 2:**
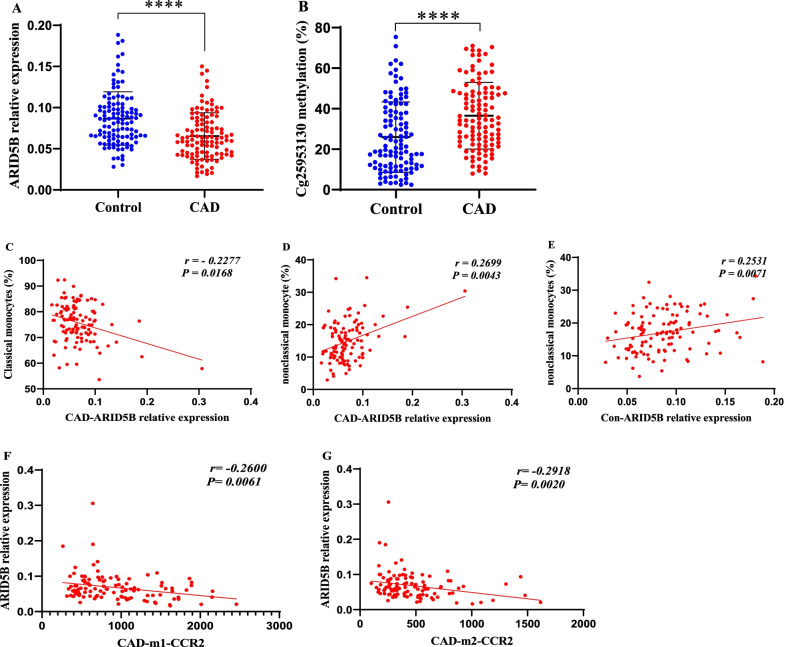
ARID5B expression is associated with monocyte subset proportions and CCR2 expression. **A** The relative mRNA expression of ARID5B in CAD patients (*n* = 110) and healthy controls (*n* = 112). **B** The methylation levels of cg25953130 in CAD patients (*n* = 110) and healthy controls (*n* = 112). **C**–**E** Spearman correlations between ARID5B expression and the proportions of monocyte subsets. **F**, **G** Spearman correlations between the expression levels of ARID5B and CCR2. m1, classical monocytes; m2, intermediate monocytes; Con, control; CAD, coronary artery disease. ****p < 0.0001

To investigate the molecular mechanism of monocyte subset shifts in CAD, we examined the expression of ARID5B in PBLs, which was recently reported to be involved in the regulation of monocyte functions [20]. The expression of ARID5B was significantly decreased in CAD patients (*p* < 0.05, Fig. 2A), whereas the methylation level of cg25953130 was significantly elevated (*p* < 0.05, Fig. 2B) and was an independent risk factor for CAD (Additional file 2: Table S3). In particular, the methylation level of cg25953130 was negatively correlated with the expression of ARID5B in the CAD (Additional file 2: Fig. S1A) and control (Additional file 2: Fig. S1B) groups. In addition, as shown in Additional file 2: Table S4, multivariate regression analysis showed that the methylation level of cg25953130 was negatively affected by age and serum HDL-C levels but was positively affected by serum TG and Hcy levels.

Spearman correlation analysis suggested that the expression level of ARID5B was negatively associated with the proportion of classical monocytes in the CAD group (*r* = − 0.2277, *p* = 0.0168, Fig. 2C) and positively associated with the proportion of nonclassical monocytes in the CAD (*r* = 0.2699, *p* = 0.0043, Fig. 2D) and control (*r* = 0.2531, *p* = 0.0071, Fig. 2E) groups. The dysregulated expression of ARID5B might be attributed to the shift in monocyte subsets. In addition, ARID5B was negatively associated with the expression of CCR2 on classical (*r* = − 0.2600, *p* = 0.0061, Fig. 2F) and intermediate (*r* = − 0.2918, *p* = 0.0020, Fig. 2G) monocytes in CAD patients. Moreover, the expression of CCR2 was related to serum Hcy (Additional file 2: Fig. S1C) and HDL-C (Additional file 2: Fig. S1D) levels.

### Folic acid reverses the suppressive effects of ox-LDL and Hcy on global DNA methylation and ARID5B expression

**Fig. 3:**
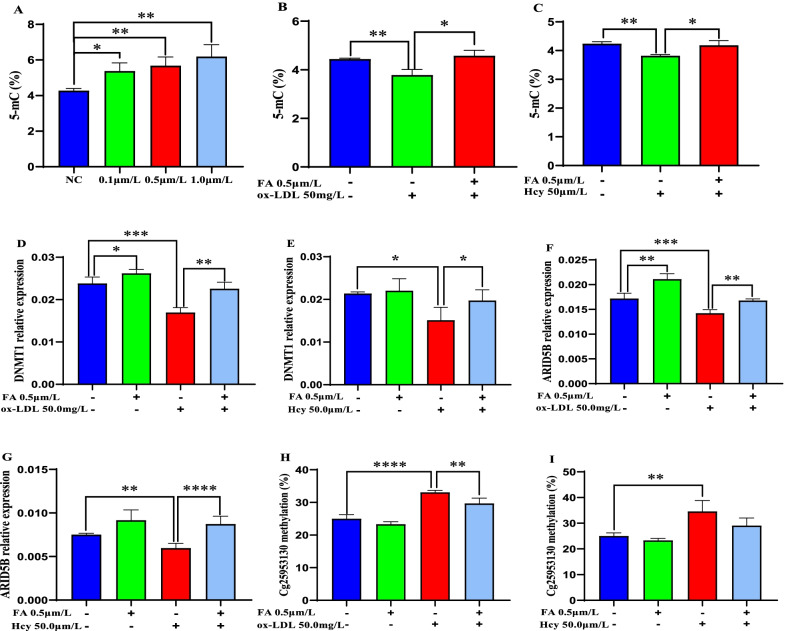
FA reverses the suppressive effects of ox-LDL and Hcy on DNA methylation and ARID5B expression. **A** The effect of FA on global 5-mC levels in THP-1 cells. **B**, **C** The effect of FA on global 5-mC levels in ox-LDL- and Hcy-treated THP-1 cells. **D**, **E** The effect of FA on the expression of DNMT1 in ox-LDL- and Hcy-treated THP-1 cells. **F**, **G** The effect of FA on the expression of ARID5B in ox-LDL- and Hcy-treated THP-1 cells. **H**, **I** The effect of FA on the methylation of cg25953130 in ox-LDL- and Hcy-treated primary monocytes. All plotted values are the mean ± SE values of at least three independent experiments. FA, folic acid; Hcy, homocysteine; ox-LDL, oxidized low-density lipoprotein. *p < 0.05, **p < 0.01, ***p < 0.001, ****p < 0.0001

THP-1 cells and primary monocytes were induced with commercial ox-LDL, Hcy, and folic acid to investigate the regulatory mechanism of lipids, Hcy, and folic acid on DNA methylation and ARID5B expression. Folic acid increased global 5-mC levels in THP-1 cells in a dose-dependent manner (*p* < 0.05, Fig. 3A), whereas in ox-LDL- and Hcy-treated THP-1 cells, global 5-mC levels (Fig. 3B, C) and the expression of DNMT1 (Fig. 3D, E) and ARID5B (Fig. 3F, G) were significantly decreased (all *p* < 0.05). Folic acid supplementation reversed the inhibitory effects of ox-LDL and Hcy on global 5-mC levels and the expression of DNMT1 and ARID5B (Fig. 3B–G). Similarly, the same effects were observed in Hcy-, ox-LDL-, and folic acid-treated primary monocytes (Additional file 2: Fig. S2).

Consistent with the positive association between TG, Hcy, and cg25953130 methylation, in ox-LDL- and Hcy-treated primary monocytes, the methylation levels of cg25953130 were significantly elevated (all *p* < 0.05), while supplementation with folic acid diminished these effects (Fig. 3H, I).

### Folic acid reverses ox-LDL- and Hcy-mediated promotion of the intermediate monocyte proportion and CCR2 expression

**Fig. 4:**
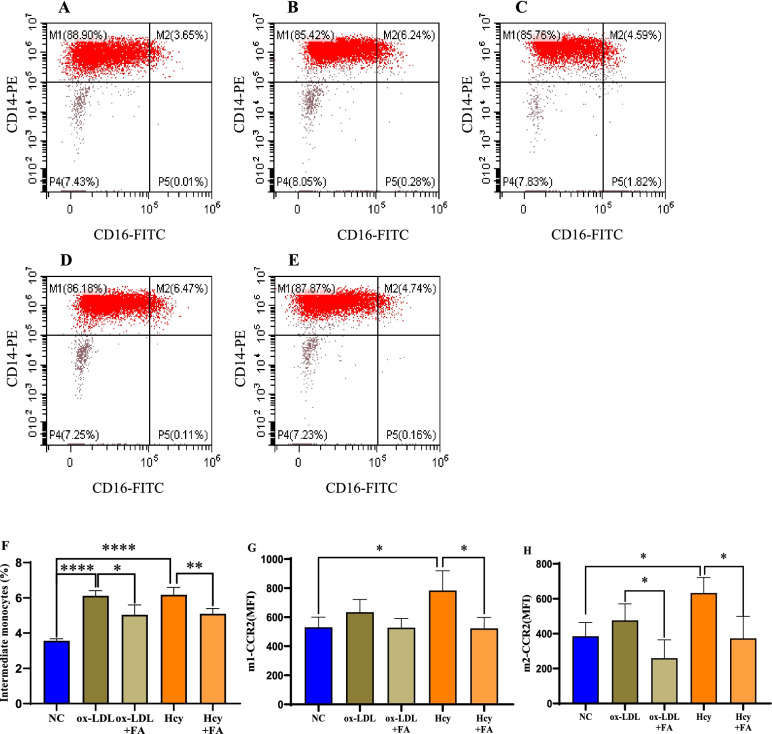
FA reverses ox-LDL- and Hcy-mediated promotion of intermediate monocyte proportions and CCR2 expression. Primary monocytes were induced using 50 μm/L ox-LDL, 50 mg/L Hcy, and 0.5 μm/L FA. **A** Negative control. **B** ox-LDL- and **C** ox-LDL + FA-treated primary monocytes. **D** Hcy- and **E** Hcy + FA-treated primary monocytes. **F** The proportion of intermediate monocytes among induced primary monocytes (%). **G** The expression level (MFI) of CCR2 on classical monocytes. **H** The expression level (MFI) of CCR2 on intermediate monocytes. All plotted values are the mean ± SE values of at least three independent experiments. m1, classical monocytes; m2, intermediate monocytes; MFI, median fluorescence intensity; NC, negative control. *p < 0.05, **p < 0.01, ****p < 0.0001

In ox-LDL- and Hcy-treated primary monocytes, the proportions of intermediate monocytes were significantly increased (*p* < 0.05, Fig. 4). Correspondingly, the expression levels of CCR2 on classical and intermediate monocytes were elevated in Hcy-treated primary monocytes but only showed increasing trends in ox-LDL-treated primary monocytes (Fig. 4G, H). Moreover, folic acid supplementation decreased ox-LDL- and Hcy-induced promotion of the proportion of intermediate monocytes and the expression of CCR2 (Fig. 4).

### ARID5B suppresses the expression of CCR2, MCP-1, and TNF-α and the migration and adhesion of THP-1 cells

**Fig. 5:**
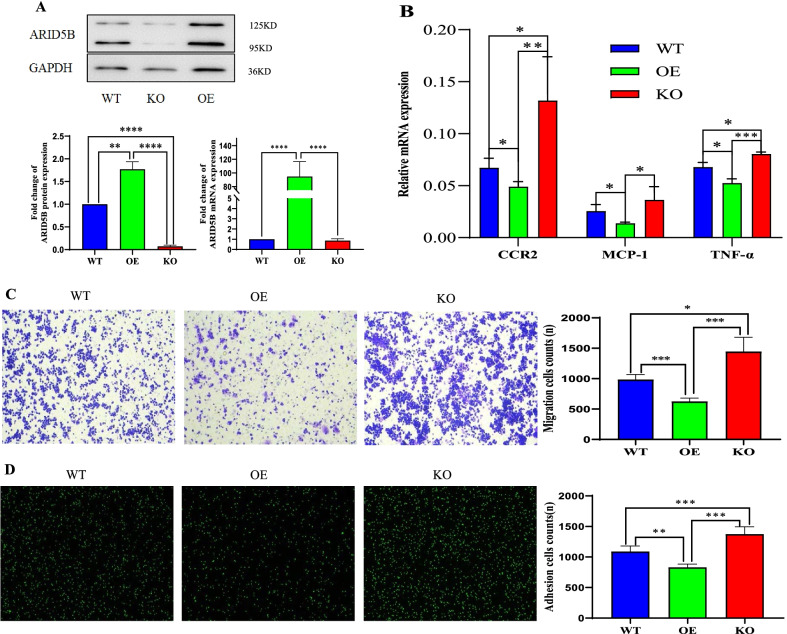
ARID5B suppresses the expression of CCR2, MCP-1, and TNF-α and the migration and adhesion of THP-1 cells. **A** The protein and mRNA expression of ARID5B in wild-type, ARID5B-overexpressing and ARID5B-knockout THP-1 cells. **B** Relative mRNA expression levels of CCR2, MCP-1, and TNF-α in THP-1 cells. **C** The migration capacity of THP-1 cells was assessed by Transwell migration assays with an inverted microscope (magnification × 100). **D** The adhesion of THP-1 cells to HUVECs was assessed using a fluorescence microscope (magnification × 100). All plotted values are the mean ± SE values of at least three independent experiments. WT, wild type; OE, overexpression; KO, knockout. *p < 0.05, **p < 0.01, ***p < 0.001, ****p < 0.0001

THP-1 cell lines with stable ARID5B overexpression and knockout were constructed to explore the effect of ARID5B on the phenotype and function of monocytes (Fig. 5A). In ARID5B-overexpressing THP-1 cells, the expression of CCR2, MCP-1, and TNF-α was significantly suppressed, whereas this effect was reversed when ARID5B expression was inhibited (Fig. 5B). Next, we simulated the adhesion of monocytes to endothelial cells and their chemotactic function in vitro. ARID5B overexpression significantly suppressed the migration (Fig. 5C) and adhesion (Fig. 5D) of monocytes; conversely, ARID5B knockout reversed these effects (Fig. 5C, D).

### ARID5B overexpression promotes M0-to-M2 polarization in macrophages and monocyte/macrophage apoptosis

**Fig. 6:**
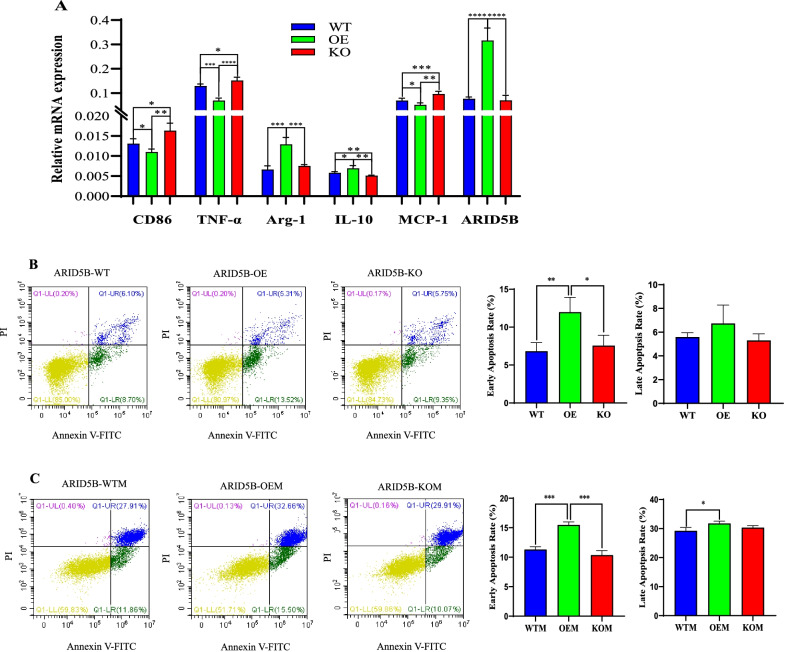
ARID5B overexpression promotes the M0-to-M2 polarization of macrophages and apoptosis in monocytes/macrophages. THP-1-derived macrophages were induced using 50 ng/mL PMA. **A** The relative mRNA expression of CD86, TNF-α, Arg-1, IL-10, MCP-1, and ARID5B in M0 macrophages. **B** The effect of ARID5B on monocyte apoptosis (%). **C** The effect of ARID5B on macrophage apoptosis (%). All plotted values are the mean ± SE values of at least three independent experiments. WTM, wild-type monocyte-derived macrophage; OEM, ARID5B-overexpressing monocyte-derived macrophage; KOM, ARID5B-knockout monocyte-derived macrophage. *p < 0.05, **p < 0.01, ***p < 0.001

M1 and M2 macrophages exert pro- and anti-inflammatory effects during AS progression, respectively. To determine the effect of ARID5B on macrophage polarization, we induced monocytes to differentiate into M0 macrophages using PMA and then examined the expression of M1 and M2 macrophage markers. ARID5B overexpression promoted the expression of M2 macrophage markers (Arg-1, IL-10) and suppressed the expression of M1 macrophage markers (CD86, TNF-α), whereas ARID5B knockout reversed these effects (Fig. 6A). This finding indicated that the reduction in ARID5B promoted the polarization of M0 macrophages to M1 macrophages. In addition, ARID5B knockout promoted the expression of MCP-1, while ARID5B overexpression induced the opposite effect (Fig. 6A).

Since depleting monocytes/macrophages in atherosclerotic plaques through apoptosis can protect against AS [25], we explored the effect of ARID5B on monocyte/macrophage apoptosis. Specifically, ARID5B overexpression promoted early apoptosis in THP-1 cells (Fig. 6B) and the derived macrophages (Fig. 6C) and facilitated late apoptosis in macrophages. On the other hand, ARID5B knockout had no significant effect on monocyte/macrophage apoptosis (Fig. 6B, C).

### Folic acid suppresses atherosclerotic plaque formation and reduces the intermediate monocyte proportion in ApoE−/− mice

In the present study, we explored the effect of folic acid on atherosclerotic plaque formation through the regulation of lipids, Hcy metabolism, and monocyte subsets. Mouse serum lipid, Hcy, and FPG levels in the different groups are shown in Additional file 2: Table S5 and Additional file 2: Fig. S3. The induction of high fat and high Hcy significantly increased serum lipids and Hcy levels in ApoE−/− mice, respectively (*p* < 0.05). Notably, folic acid supplementation significantly reduced serum lipids and Hcy levels (*p* < 0.05).

**Fig. 7:**
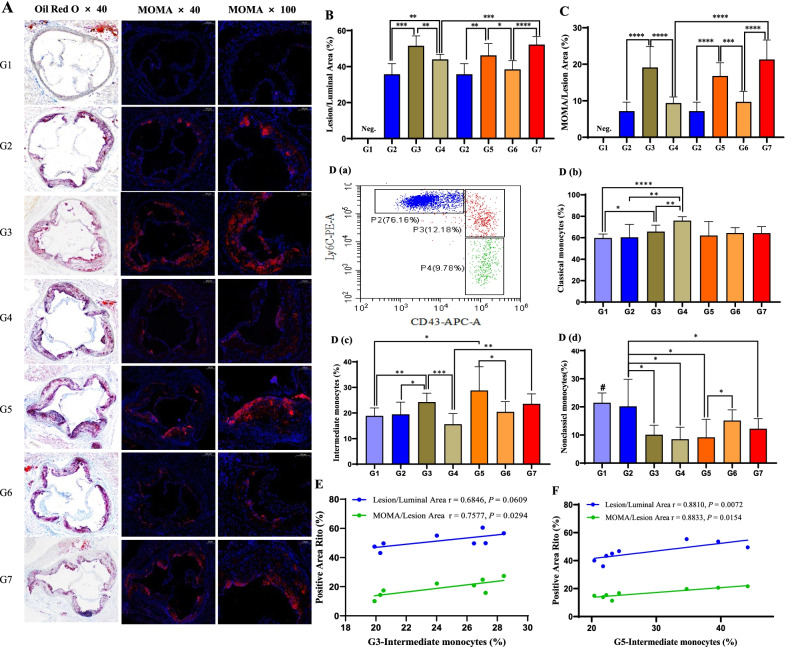
FA suppresses AS plaque formation and reduces the intermediate monocyte proportion in ApoE−/− mice. Mice were divided into seven subgroups: G1, ApoE−WT + ND; G2, ApoE−/− + ND; G3, ApoE−/− + HFD; G4, ApoE−/− + HFD + FA; G5, ApoE−/− + ND + Hcy; G6, ApoE−/− + ND + Hcy + FA; and G7, ApoE−/− + HFD + Hcy + FA. **A** Representative oil red O-stained and MOMA-immunostained images of G1–G7 mouse aortic sinus cross sections. For immunostaining, cell nuclei were stained with DAPI (blue) and monocytes/macrophages were stained with MOMA antibodies (red). **B** The severity of vascular lesions was evaluated by determining the ratio of the atherosclerotic plaque lesion area to the luminal area (%). **C** The accumulation of monocytes/macrophages in atherosclerotic plaques was evaluated by determining the ratio of the MOMA-positive area to the atherosclerotic plaque lesion area (%). **D** Mouse monocyte subset gating strategy (**a**) and the proportions of classical (**b**), intermediate (**c**), and nonclassical (**d**) monocytes. **E**, **F** Correlation analysis of the proportion of intermediate monocytes and the severity of vascular lesions (blue) and the accumulation of monocytes/macrophages (green) in high fat (G3)- and high Hcy (G5)-treated ApoE−/− mice. Neg., no AS plaque formation; WT, wild type; ND, normal diet; HFD, high-fat diet; FA, folic acid; Hcy, homocysteine. #, the G1 group was significantly different from the other six groups; *p < 0.05, **p < 0.01, ***p < 0.001, ****p < 0.0001

Next, we evaluated the formation of atherosclerotic plaques in the aortic sinus of ApoE−/− mice by oil red O staining. After 27 weeks, no atherosclerotic plaques had formed in wild-type ApoE mice fed a normal diet (G1), but extensive atherosclerotic plaques formed in all ApoE−/− mice (G2-7, Fig. 7A). In particular, the induction of high fat (G3) and high Hcy (G5) significantly aggravated (*p* < 0.05) the formation of atherosclerotic plaques, whereas folic acid supplementation in mice induced with high fat (G4) and high Hcy (G6) reduced these effects (Fig. 7B). We further assessed the accumulation of monocytes/macrophages in atherosclerotic plaques by immunofluorescence staining with MOMA antibodies. Consistent with atherosclerotic plaque formation, high fat and high Hcy induction promoted monocyte/macrophage accumulation in atherosclerotic plaques; similarly, folic acid supplementation reduced these effects (Fig. 7C).

We next investigated the effects of high lipids, Hcy, and folic acid on the shift in monocyte subsets. As shown in Fig. 7D, the proportions of classical monocytes [Fig. 7D (b)] were increased in high fat-induced mice. The proportions of intermediate monocytes [Fig. 7D (c)] were increased in high fat- and high Hcy-induced mice, and folic acid supplementation reversed these effects. The proportions of nonclassical monocytes [Fig. 7D (d)] were dramatically reduced in high fat- and high Hcy-induced mice. In high fat- and high Hcy-induced mice, the proportions of intermediate monocytes were positively associated with the severity of atherosclerotic plaque lesions and monocyte/macrophage accumulation (Fig. 7E, F), suggesting a AS promotion effect of intermediate monocytes.

However, in high fat- and high Hcy-co-induced ApoE−/− mice (G7), folic acid-mediated reversal of lipid and Hcy disorders, AS plaque formation, monocyte/macrophage accumulation, and monocyte subset shifts were not dominant (Fig. 7A–D).

### Folic acid reverses the suppressive effects of lipids and Hcy on global DNA methylation and ARID5B expression in mice

**Fig. 8:**
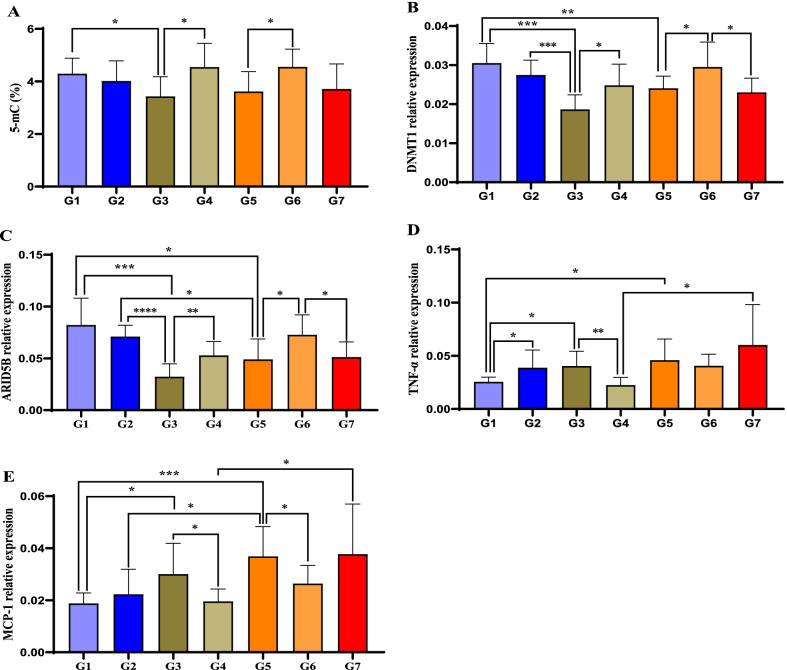
FA reverses the suppressive effects of lipids and Hcy on global DNA methylation and ARID5B expression in mice. The effect of FA on global 5-mC levels (**A**) and the expression of DNMT1 (**B**), ARID5B (**C**), TNF-α (**D**), and MCP-1 (**E**) in mice. *p < 0.05, **p < 0.01, ***p < 0.001, ****p < 0.0001

Finally, we assessed the regulatory effects of lipids, Hcy, and folic acid on global DNA methylation and ARID5B expression. Consistent with our clinical and in vitro data, high fat and high Hcy induction reduced global 5-mC levels (Fig. 8A), suppressed the expression of DNMT1 (Fig. 8B) and ARID5B (Fig. 8C), and promoted the expression of TNF-α (Fig. 8D) and MCP-1 (Fig. 8E). Conversely, folic acid supplementation reversed these effects on DNA methylation and gene expression in groups that were induced individually with high fat and high Hcy but did not affect high fat- and Hcy-co-induced ApoE−/− mice.

## Discussion

This study revealed that folic acid alleviates the atherogenic function of the increased intermediate monocytes induced by HL and HHcy through the DNA methylation pathway, which includes global DNA hypomethylation and ARID5B hypermethylation.

We first revealed that intermediate monocytes were elevated in CAD patients, had the lowest global 5-mC levels, and were negatively associated with PBLs’ global 5-mC levels. The DNA methylation changes, including decreased global 5-mC levels and hypermethylated cg25953130, which suppressed ARID5B expression, could be induced by HL and HHcy. Furthermore, reduced ARID5B expression might mediate the increased proportion of intermediate monocytes by regulating the expression of CCR2 and TNF-α, monocyte adhesion and migration, and apoptosis. Finally, we found that folic acid could reverse global DNA demethylation and cg25953130 hypermethylation, reduce the proportion of intermediate monocytes, and hinder the progression of AS, which might be the underlying mechanism for the protective effects of folic acid.

### Global DNA hypomethylation and ARID5B hypermethylation might inhibit the differentiation of monocyte subsets and inhibit AS inflammation

Transcriptomic and epigenetic approaches have characterized the heterogeneity of monocytes into three distinct subsets [13, 26], among which classical monocytes originate from bone marrow and gradually differentiate into intermediate and nonclassical monocytes in circulation [27]. To the best of our knowledge, this is the first study to report significant differences in global 5-mC levels among the three monocyte subsets based on direct evidence, and intermediate monocytes were significantly increased and had the lowest global 5-mC levels in CAD patients. This result might be consistent with Zawada’s findings that suggested that intermediate monocytes had more hypomethylated CpG loci than the other two subsets; pathway analysis also indicated that the differentially methylated genes were linked to monocyte differentiation [13]. In addition, a recent study suggested that ten-eleven translocation-3 (TET3) DNA demethylase knockout could promote the differentiation of classical monocytes into more mature intermediate and nonclassical monocytes [28], suggesting the effect of global DNA hypomethylation on monocyte subset shifts. Therefore, we hypothesized that the DNA hypomethylation induced by HL and HHcy might disturb the differentiation of monocyte subsets, thereby leading to increased proportions of intermediate monocytes, which, in turn, decreased global 5-mC levels in PBLs in CAD patients. Moreover, the decreased global 5-mC levels in PBLs could be a marker of inflammation in CAD patients since intermediate monocytes are considered inflammatory monocytes due to their correlations with increased reactive oxygen species (ROS) [4] and the inflammatory cytokine TNF-α in AS [29].

Aberrant global and local CpG DNA methylation are involved in AS pathogenesis [15, 23, 24, 30]. DNA hypomethylation in the CD40 promoter enhances the generation of CD40 + inflammatory intermediate monocytes [15], revealing that certain gene methylation variations could regulate the monocyte functional phenotype. This finding prompted us to investigate whether a similar epigenetic mechanism was involved in the regulation of monocyte subsets. Interestingly, we found the following evidence to suggest that ARID5B might be involved in monocyte subset regulation: (i) HL and HHcy could trigger cg25953130 hypermethylation and decrease ARID5B expression in CAD patients and ox-LDL- and Hcy-treated primary monocytes; (ii) ARID5B was associated with increases in nonclassical and decreases in classical monocyte proportions; and (iii) ARID5B was negatively associated with the expression of CCR2, which was upregulated in intermediate monocytes. Since the expression of CCR2 gradually declined during the differentiation of monocyte subsets [27], we hypothesized that the elevated expression of CCR2 on intermediate monocytes might reflect the inhibition of the differentiation of intermediate monocytes to nonclassical monocytes. ARID5B has been reported to be involved in differentiation regulation in skeletal muscle, smooth muscle, and cartilage cells as a member of the AT-rich interaction domain (ARID) protein family [31–33].

### ARID5B regulates monocyte cytokine expression, adhesion, migration, macrophage polarization, and apoptosis

We further examined the regulatory effects of ARID5B on monocyte functional phenotypes. The recruitment of circulating monocytes to atherosclerotic plaques and their adhesion to vascular endothelial cells mainly rely on CCR2 [34]. In ARID5B-overexpressing THP-1 cells, the expression of CCR2 was reduced, and the inhibitory effect of ARID5B on the expression of CCR2 was confirmed in our subsequent migration and adhesion experiments. These results suggested that blocking the ARID5B-CCR2 pathway might be helpful for AS prevention [35]. The antiatherosclerotic effect of ARID5B was also reflected in the inhibition of the expression of chemokines (MCP-1) and inflammatory (TNF-α) factors in monocytes and macrophages. Monocyte/macrophage apoptosis provides a homeostatic mechanism to regulate inflammation by eliminating activated and differentiated monocytes/macrophages [36]. In addition, intermediate monocytes are more susceptible to spontaneous apoptosis than classical subsets [37]. Our data revealed that ARID5B overexpression promoted the polarization of M0 macrophages to M2 macrophages and apoptosis in monocytes/macrophages, which might be another mechanism by which ARID5B regulates the phenotype of monocytes/macrophages to protect against AS. We hypothesized that ARID5B facilitated an improper inflammatory response and pathological progression in AS by promoting monocyte/macrophage apoptosis and inhibiting inflammatory macrophage polarization; however, this mechanism needs to be further explored.

### Folic acid might exert its antiatherosclerotic effects through monocyte subset shifts mediated by global DNA remethylation and ARID5B upregulation

Studies have shown that folic acid can reduce CVD risk [38]. The beneficial effect of folic acid on DNA methylation is partially attributed to its role as a methyl group donor in Hcy remethylation [39]. In vivo, approximately 50% of Hcy is remethylated to form methionine [40]. Herein, we confirmed the Hcy-lowering effect of folic acid in Hcy-treated ApoE−/− mice. Moreover, a recent study revealed that folic acid supplementation could normalize Hcy levels, reduce SAH concentrations, and reverse aberrant DNA methylation in vascular endothelial cells [41]. This study further confirmed folic acid-mediated reversal of Hcy-induced global DNA hypomethylation in monocytes.

In addition to HHcy, HL can inhibit the expression of DNMTs, leading to DNA hypomethylation [24]. Folic acid was reported to reduce lipid levels in postmenopausal Korean women with T2DM [42]. Our animal model also revealed the serum lipid-lowering effect of folic acid. Notably, folic acid was shown to increase DNMT1 activity, expression, and genomic 5-mC levels in ox-LDL-treated HUVECs [43]. Similarly, our data revealed the same effect of folic acid on ox-LDL-treated monocytes.

It is worth mentioning that the proportion of intermediate monocytes was associated with the severity of atherosclerotic plaque lesions and monocyte/macrophage accumulation, revealing that intermediate monocytes promote AS. Folic acid might mediate the decrease in the proportion of intermediate monocytes by restoring the decrease in ARID5B expression and global DNA hypomethylation, exerting an antiatherosclerotic effect on ApoE−/− mice.

Interestingly, unlike global DNA hypomethylation induced by HL and HHcy, the methylation level of cg25953130 was upregulated, which was inconsistent with the decreased expression of DNMT1. Three DNMTs (DNMT1, DNMT3A, and DNMT3B) with distinct functions are involved in the generation and maintenance of DNA methylation patterns [44]. Liu et al*.* [45] found that DNMT3B mediated CREG gene hypermethylation, which contributed to endothelial dysfunction, revealing the multiple DNA methylation patterns mediated by different DNMTs. We hypothesized that DNMT1 did not control the methylation of cg2595310, and the mechanism needs to be further explored. Notably, folic acid exerted an inhibitory effect on cg25953130 methylation in primary monocytes. However, it should be pointed out that the methylation patterns of cg25953130 between cases and controls in our study are the opposite of those in Liu et al*.* [20], which may be attributed to the differences in clinical parameters of the enrolled subjects, sample species, and inclusion criteria for the enrolled subjects.

This study has a few limitations. Although we found that ARID5B could regulate the expression of CCR2 and was associated with monocyte subsets, direct evidence of ARID5B-mediated regulation of monocyte subsets needs to be further confirmed in animal studies. In addition, the regulatory effect of cg25953130 methylation on ARID5B expression needs to be further explored.

## Conclusion

Our study suggested that HL and HHcy decreased global DNA methylation and ARID5B expression, which might mediate the increased proportion of intermediate monocytes. Moreover, we discovered that folic acid could reverse these processes and inhibit the formation of atherosclerotic plaques, which might have the preventive and therapeutic value for atherosclerosis.

## Materials and methods

### Study population

From November 2018 to July 2019, 180 CAD patients and 210 healthy controls were recruited from Zhongnan Hospital of Wuhan University (Hubei, China) based on the previous inclusion and exclusion criteria [46]. Clinical characteristics, such as a history of type 2 diabetes mellitus (T2DM) and hypertension, fasting plasma glucose (FPG), lipid and Hcy levels, and leukocyte counts, were collected.

This study was approved by the Medical Ethics Committee of Zhongnan Hospital of Wuhan University (Approval number, 2018017, Wuhan, China) and was conducted in accordance with the Declaration of Helsinki. All participants signed informed consent forms.

### Cell culture

THP-1 cells and human umbilical vein endothelial cells (HUVECs) were obtained from the American Type Culture Collection (ATCC, Manassas, USA). THP-1 cells were cultured in RPMI 1640 medium (11875093, Gibco, CA, USA) supplemented with 10% (v/v) fetal bovine serum (FBS, 10099141, Gibco) and 1% (v/v) PS (100 IU/mL penicillin G & 100 IU/mL streptomycin, C0222, Beyotime, Shanghai, China). HUVECs were cultured in DMEM high glucose medium (11965092, Gibco) supplemented with 10% (v/v) FBS and 1% (v/v) PS. CD14 + primary monocytes were obtained from peripheral blood through magnetic bead sorting and were cultured in RPMI 1640 medium supplemented with 15% (v/v) FBS and 1% (v/v) PS. All cells were maintained in a humidified atmosphere containing 5% CO_2_ at 37 °C.

THP-1 cells (8 × 10^5^ cells/mL) in the treatment group were exposed to 2 mL of medium containing 50 mg/L ox-LDL (YB-002, Yiyuan Biotech, Guangzhou, China); 50 μmol/L Hcy (H4628, Sigma-Aldrich, Darmstadt, Germany); 0.1, 0.5, or 1.0 μmol/L FA (F8758, Sigma-Aldrich); 50 mg/L ox-LDL plus 0.5 μmol/L FA; or 50 μmol/L Hcy plus 0.5 μmol/L FA for 72 h. (The medium and induction reagents were changed every 24 h.) CD14 + primary monocytes (6 × 10^5^ cells/mL, 2 mL) were induced and cultured for 12 h using the same grouping method. Cells that were cultured in RPMI 1640 medium without induction reagents were used as a negative control.

### ARID5B overexpression and knockout in THP-1 cells

The pGH125-ARID5B (NM_032199.3)-puro plasmid was constructed to induce overexpression. The ARID5B knockout plasmid was constructed using the CRISPR/Cas9 method. In brief, the unique single-guide RNA (gRNA) sequence (sgRNA sequence: 5’-gcagaccccaaaggtccttg-3’) of the ARID5B gene was designed and cloned into the lentiCRISPR v1 plasmid. To generate stable cell lines, HEK293 cells were cotransfected with pGH125-ARID5B or lentiCRISPR v1 plasmid and packaging plasmids using a transfection reagent (6366244001, Roche, USA) according to the manufacturer's instructions. After 72 h of culture, the viral supernatant was collected and used to infect THP-1 cells (1 × 10^5^ cells/mL). After 72 h, THP-1 cells were selected using 0.3 μg/ml puromycin. Puromycin-resistant cells were then seeded into 96-well plates for clonal expansion and analyzed using qPCR and western blotting.

### Animals, diets, and experimental procedures

Apolipoprotein E-deficient (ApoE−/−) mice have a homozygous deletion of the ApoE gene that was prepared by homologous recombination technology. This mouse model exhibits abnormal hyperlipidemia and can develop atherosclerosis under spontaneous or induced conditions. ApoE−/− mice have extensively been used to study the mechanisms underlying the initiation and progression of atherosclerosis. In this study, 8 wild-type and 48 ApoE−/− male C57BL/6 J mice aged 5 weeks were purchased from Charles River Laboratory Animal Technology Co., Ltd. (Beijing, China) in November 2019. All animals were housed in a pathogen-free facility with a temperature of 24 ± 1 °C, a relative humidity of 50 ± 1%, and a light/dark cycle of 12/12 h. All animal studies (including the mouse euthanasia procedure) were performed in compliance with the institutional animal care regulations and guidelines of Wuhan University and were conducted according to the AAALAC and IACUC guidelines (IACUC approval number, 2019174).

The wild-type control group was fed a normal diet (G1, WT + ND), and 48 ApoE−/− mice were randomly distributed into six groups (8 per group): (1) normal diet (G2, ApoE−/− + ND), (2) high-fat diet (G3, ApoE−/− + HFD), (3) high-fat diet + 1.6 μg/mL FA (F8758, Sigma-Aldrich) daily in drinking water (G4, ApoE−/− + HFD + FA), (4) normal diet + 1.8 g/L Hcy (H4628, Sigma-Aldrich) daily in drinking water (G5, ApoE−/− + ND + Hcy), (5) normal diet + 1.6 μg/mL FA & 1.8 g/L Hcy daily in drinking water (G6, ApoE−/− + ND + FA + Hcy), and (6) high-fat diet + 1.6 μg/mL FA & 1.8 g/L Hcy daily in drinking water (G7, ApoE−/− + HFD + FA + Hcy). Drinking water containing FA and Hcy was changed every 3 days.

The Western-type high-fat diet (20% fat, 1.25% cholesterol) was purchased from Peking Huafukang Laboratory Animal Center (Beijing, China). Mice without special treatment were given normal maintenance feed and normal drinking water. After 27 weeks, all mice were fasted overnight before ether anesthesia, after which blood and tissue samples were immediately collected and stored at −80 °C.

### Macrophage polarization

To evaluate the regulatory effect of ARID5B on THP-1-derived macrophage polarization, the stable THP-1 cells (6 × 10^5^ cells/mL, 2 mL) were induced to form M0 macrophages using 50 ng/mL PMA (P1585, Sigma-Aldrich) and cultured for 24 h for mRNA expression analysis or were further cultured for 48 h with new medium without PMA for apoptosis analysis.

### DNA extraction, enzymatic digestion, and global 5-mC level detection

Genomic DNA was extracted using the phenol/chloroform method or a commercial DNA extraction kit (3001050, Simgen, Hangzhou, China) based on the sample size and was quantified by a NanoDrop 2000 (Thermo Scientific, Wilmington, USA). The enzymatic digestion of genomic DNA and the analysis of global 5-mC levels were performed according to our previously described ultrahigh-performance liquid chromatography–mass spectrometry/mass spectrometry (UPLC–MS/MS) method [24].

### RNA extraction and RT–qPCR

Total RNA was extracted using TRIzol reagent (15596026, Invitrogen, CA, USA) or a commercial RNA extraction kit (5100050, Simgen) based on the sample size and was quantified by a NanoDrop 2000 (Thermo Scientific). Total RNA was reverse-transcribed into cDNA using a commercial reversal kit (FSQ-301, TOYOBO, Osaka, Japan). Real-time quantitative polymerase chain reaction (RT–qPCR) was performed using a Bio-Rad CFX96 real-time system (Bio-Rad, CA, USA). The relative expression levels of target genes were calculated using the comparative crossing threshold method of relative quantification (^△^Cq) and are expressed as fold change values. GAPDH was used as an internal reference gene. Each sample was analyzed at least three times, and the detailed primer information is shown in Additional file 2: Table S6.

### Flow cytometry

Approximately 100 μL of human and mouse whole blood samples was used for monocyte subset analysis, which was performed according to our previously described method [19]. In brief, human blood samples were stained with a mixture of four mouse anti-human monoclonal fluorochrome-conjugated antibodies (anti-CD14-PE, 5 μL; anti-CD16-FITC, 10 μL; anti-CCR2-APC, 5 μL; anti-CD86-BV421, 2 μL) (55398, 555406, 558406, 562433, BD PharMingen, NJ, USA); mouse blood samples were stained with a mixture of three rat anti-mouse monoclonal fluorochrome-conjugated antibodies (anti-Ly6C-PE, 5 μL; anti-CD43-APC, 5 μL; anti-CD115-Alexa Fluor ® 488, 5 μL) (560592, 560663, 135512, BD PharMingen). Approximately 100 μL of cultured human CD14 + primary monocytes was stained with a mixture of three mouse anti-human monoclonal fluorochrome-conjugated antibodies (anti-CD14-PE, 5 μL; anti-CD16-FITC, 5 μL; anti-CCR2-APC, 5 μL) (BD PharMingen). The expression level of CCR2 was quantified by flow cytometry and was expressed as the median fluorescence intensity (MFI). Fluorescence was standardized using multiple peaks rainbow calibration beads (Spherotech, Chicago, USA) to ensure the reproducibility and comparability of the median fluorescence intensity (MFI) throughout the study period, as previously described [47]. Flow cytometry was performed using a Beckman CytoFLEX flow cytometer (Beckman Coulter, CA, USA). The gating strategy for human and mouse monocyte subsets is shown in Additional file 2: Fig. S4 and Fig. S5, respectively.

### Monocyte subset sorting

Three monocyte subsets were sorted from 15 CAD patients and 8 healthy volunteers using a BD FACSAria III (BD PharMingen). In brief, 10 mL of EDTA-anticoagulated whole blood samples were collected, and red blood cells were lysed using 1 × RBC lysis buffer (555,899, BD PharMingen). White blood cells were stained with a mixture containing three mouse anti-human monoclonal fluorochrome-conjugated antibodies (anti-CD14-PE, 200 μL; anti-CD16-FITC, 400 μL; anti-CD86-APC, 50 μL) (555398, 555406, 555660, BD PharMingen). Monocytes were gated in an SSC/CD86 + dot plot, and then, based on CD14 and CD16 expression, CD86 + monocytes were divided into classical, intermediate, and nonclassical subsets. Based on the gating strategy, the three monocyte subsets were sorted separately. Flow cytometry was used to reanalyze the obtained monocytes, and the results indicated that the sorted monocyte subsets had good purity (Additional file 2: Fig. S6).

### Isolation of primary monocytes

Approximately 50 mL of fresh peripheral blood was collected from healthy volunteers, and sodium citrate was used for anticoagulation. First, peripheral blood mononuclear cells (PBMCs) were isolated by density gradient centrifugation using 1.077 g/mL Ficoll/Hypaque (P4350, Solarbio, Beijing, China). Then, monocytes were purified from PBMCs using the positive selection method with a magnetic-activated cell sorter (MACS) system (130-042-201, Miltenyi, Bergisch Gladbach, Germany) and CD14 microbeads (130-050-201, Miltenyi) according to the manufacturer’s instructions. Flow cytometry was used to identify the purity of the obtained monocytes, which were labelled with CD14 monoclonal fluorochrome-conjugated antibodies (555398, BD PharMingen). Monocyte purity was at least 90% (Additional file 2: Fig. S7).

### MDRE–qPCR assay

A methylation-dependent restriction enzyme digestion-based qPCR (MDRE**–**qPCR) assay was used to examine the methylation level of the ARID5B gene cg25953130 CpG site. DNA samples (including sample DNA and positive and negative methylation controls) were first diluted to 40 ng/μL with ultrapure water. The diluted DNA samples were digested using a 10 μL mixture of 5 μL of DNA, 0.4 μL of FspEI (R0662S, NEB, Beijing, China), 1 μL of buffer, 0.2 μL of activator, and 3.4 μL of ddH_2_O. Another 10 μL mixture (5 μL of DNA, 1 μL of buffer, 0.2 μL of activator, and 3.8 μL of ddH_2_O) without enzymatic digestion was used as a negative control. The reaction mixtures were incubated at 37 °C for 3 h and then incubated at 80 °C for 20 min for enzyme inactivation. The cg25953130 methylation level was measured using a 10 μL mixture including 1 μL of enzyme-digested product (or negative control), 5 μL of 2 × T5 Fast qPCR Mix (TSE202, Tsingke, Beijing, China), 1 μL of forward primer (5’-GAATTGGAATAGCGCCAGGT-3’), 1 μL of reverse primer (5’-AAGGAAATATGAATGTG CTCACG-3′), and 3.2 μL of ddH_2_O. The reaction mixtures were incubated at 95 °C for 1 min, followed by 40 cycles (denaturation, 95 °C for 10 s; annealing, 58 °C for 5 s; extension, 72 °C for 15 s). The efficiency of the restriction digestion was evaluated as follows: The methylation level of the unmethylated negative control should approach 0%, and the methylated positive control should approach 100%. Otherwise, the samples were redigested. The formula for calculating the methylation level was as follows: cg25953130 methylation level (%) = 100% × [1 − 2^△^Cq (control − digestion) group].

### HUVEC adhesion assay

One day prior to the adhesion assay, 1 mL of HUVECs was seeded into 12-well plates at a density of 5 × 10^5^ cells/mL and cultured in 5% CO_2_ at 37 °C until the cells reached 90% confluence. In the meantime, 1 mL of THP-1 cells (1 × 10^6^ cells/mL) was stained with 1 μL of the fluorescent probe BCECF-AM (5 mM) (Beyotime, Shanghai, China) in the dark for 30 min. Then, the cells were washed twice using phosphate-buffered saline (PBS, pH = 7.4, P1022, Solarbio). After being resuspended, 1 mL (5 × 10^5^ cells/mL) of the stained THP-1 cells was added to the monolayer formed by the HUVECs. After the cells were incubated at 37 °C for 60 min, the nonadherent cells were washed away using PBS. Under an inverted fluorescence microscope, five fields per well were randomly selected and photographed at 100 × magnification to calculate the number of adherent THP-1 cells. Image-Pro Plus (v 6.0) software was used for image analysis.

### Transwell assay

THP-1 chemotaxis in response to MCP-1 was evaluated with a Transwell chamber with a pore size of 8 μm (CLS3422, Corning, Toledo, USA). Approximately 0.6 mL of RPMI 1640 medium containing 200 ng/mL MCP-1 (SRP3109, Sigma-Aldrich) and 5% (v/v) FBS was first added to the well of a 24-well plate. Then, 0.2 mL of THP-1 cells (1 × 10^6^ cells/mL) was added to the Transwell chamber. The chamber was placed into the well and incubated at 37 °C for 60 min. PBS (pH = 7.4) was used to wash away the medium, and the cells on the upper side of the filters were washed twice. The cells on the underside of the filters were fixed with 1.0 mL of 4% paraformaldehyde (BL539A, Biosharp, Hefei, China) for 10 min and then stained with 0.6 mL of 0.1% crystal violet staining solution (94,448, Sigma-Aldrich) for 10 min. Under an inverted microscope, five fields per chamber were randomly selected and photographed at 100 × magnification to calculate the number of migrated THP-1 cells. Image-Pro Plus (v 6.0) software was used for image analysis.

### Apoptosis assay

A commercial Annexin V-FITC/PI Apoptosis Kit (BB-4101, BestBio, Shanghai, China) was used to evaluate the early and late apoptosis rates according to the manufacturer's instructions. In brief, 0.5 mL of cultured cells (1 × 10^6^ cells/mL) was first stained with 5 μL of Annexin V-FITC in the dark for 15 min and then stained with 5 μL of PI for 5 min. The apoptosis rate was immediately analyzed using flow cytometry.

### Western blotting

Cells were lysed in ice-cold RIPA lysis buffer (R0020, Solarbio), and protein levels were quantified using a BCA protein assay kit (P0012S, Beyotime). The extracted cellular proteins were mixed with loading buffer (v/v = 4:1) (P1040, Solarbio) and boiled for 10 min. Protein samples (30 μg per well) were separated by 8% SDS–PAGE gels and transferred to PVDF membranes. The membranes were blocked at room temperature with 5% BSA in 1 × TBST (0.2% Tween-20, pH = 7.6) buffer for 2 h and then incubated with primary antibodies against GAPDH (1:100,000, A19056, ABclonal, Wuhan, China) and ARID5B (1:2000, NBP1-83622, Novus, Shanghai, China) at 4 °C overnight. After being washed with 1 × TBST buffer four times (10 min each time), the membranes were then incubated with HRP-conjugated mouse anti-rabbit (1:5000, AS061, ABclonal) and goat anti-mouse (1:5000, AS003, ABclonal) secondary antibodies at room temperature for 1 h. Protein bands were visualized by chemiluminescence using an ECL reagent (PE0010, Solarbio) and photographed by a Tanon-5200 Chemiluminescent Imaging System (Tanon, Shanghai, China). Image-Pro Plus (v 6.0) software was used to perform the densitometric semiquantitative analysis of protein bands. The protein expression was normalized against GAPDH.

### Assessment of atherosclerotic plaques

Atherosclerotic plaque formation in the mouse aortic sinus was assessed using oil red O staining (O0625, Sigma-Aldrich). Heart samples were embedded in OCT (4583, Sakura, USA) compound and frozen in liquid nitrogen. The frozen tissue was thawed at − 20 °C for 30 min, after which the aortic sinus was sectioned into consecutive 6-μm-thick sections at − 20 °C. Aortic sinus sections with three complete aortic valves were selected for oil red O staining and photographed using a microscope (Nikon, Tokyo, Japan) at 40 × magnification. The degree of atherosclerotic lesions was evaluated by determining the ratio of atherosclerotic plaque areas to vessel lumen areas, which were quantified using Image-Pro Plus (v 6.0) software.

### Immunofluorescence staining

Frozen aortic sinus sections were dried and fixed in cold acetone for 10 min and were repaired using sodium citrate–EDTA. Aortic sinus sections were then washed with PBS buffer (pH = 7.4) three times and blocked with 2% BSA for 1 h at 37 °C. Then, the sections were incubated with rabbit anti-mouse MOMA (Monocyte & Macrophage) (1:50, ab33451, Abcam, Shanghai, China) primary antibodies at 4 °C overnight and with Alexa Fluor® 488-conjugated goat anti-rabbit (1:200, ab150077, Abcam) secondary antibodies for 50 min at 25 °C. After being washed with PBS buffer three times, the sections were incubated with DAPI (62248, Thermo Scientific). After the sections were washed and slightly dried, they were mounted with an anti-fluorescence quenching mounting medium. A Nikon upright fluorescence microscope (Nikon) was used to observe and obtain fluorescence images. The positive areas of the sections were quantified using Image-Pro Plus (v 6.0) software.

### Statistical analysis

Based on their distributions, continuous variables are presented as the mean ± SD or as the median (interquartile range). Comparisons between continuous variables were performed using Student’s* t* test or the Mann–Whitney *U* test. Categorical variables are presented as frequencies (n, %), and the chi-square test was used to evaluate the significance of any differences between groups. Spearman or Pearson bivariate correlation analyses were used to analyze the correlations in the present study. Multivariate regression analysis was used to evaluate the effects of CAD risk factors on the methylation level of cg25953130. SPSS software version 17.0 (SPSS, Chicago, USA) and GraphPad Prism 8.0 (GraphPad, San Diego, USA) were used for statistical analyses. A *p* < 0.05 (two-tailed) was considered to be statistically significant.

## Supplementary Information

**Additional file 1:** Original datasets.

**Additional file 2: Table S1.** Clinical characteristics of the enrolled subjects. **Table S2.** Clinical characteristics of the randomly selected subjects. **Table S3.** Independent risk factors for CAD. **Table S4.** Bivariate and multivariate association between clinical parameters and the methylation levels of cg25953130. **Table S5.** Serum lipids, Hcy and FPG levels of mouse. **Table S6.** Primers for RT-qPCR. **Figure S1.** Spearman correlation analysis. **Figure S2.** The effects of Hcy, ox-LDL and FA on DNMT1 and ARID5B expression in primary monocytes. **Figure S3.** The effect of folic acid on the lipid and Hcy metabolism in mice. **Figure S4.** Gating strategy for human monocyte subsets. **Figure S5.** Gating strategy for mouse monocyte subsets. **Figure S6.** Gating strategy for monocyte subsets sorting and purity identification. **Figure S7.** Purity identification for CD14+ monocytes.

## Data Availability

All data generated or analyzed during this study are included in this published article [and its supplementary files].

## Abbreviations

CAD: Coronary artery disease
5-mC: 5-Methylcytosine
ARID5B: Transcription coactivator for histone H3K9me2 demethylation
FCM: Flow cytometry
PBLs: Peripheral blood leukocytes
ox-LDL: Oxidized low-density lipoprotein
Hcy: Homocysteine
FA: Folic acid
HHcy: Hyperhomocysteinemia
MFI: Median fluorescence intensity
FPG: Fasting plasma glucose
TC: Total cholesterol
TG: Triglyceride
LDL-C: Low-density lipoprotein—cholesterol
HDL-C: High-density lipoprotein—cholesterol
HUVECs: Human umbilical vein endothelial cells
PBMC: Peripheral blood mononuclear cell
CI: Confidence interval
OR: Odds ratio
UPLC–MS/MS: Ultrahigh-performance liquid chromatography–mass spectrometry/mass spectrometry
HL: Hyperlipidemia
