# Supplementary material for: Atheroprotective mechanism by which folic acid regulates monocyte subsets and function through DNA methylation
Source: Clin Epigenetics. 2022 Feb 28;14:32. doi: 10.1186/s13148-022-01248-0 (PMC8887029; doi:10.1186/s13148-022-01248-0)
Supplement: Supplementary file 2 — Additional file 2: Table S1. Clinical characteristics of the enrolled subjects. Table S2. Clinical characteristics of the randomly selected subjects. Table S3. Independent risk factors for CAD. Table S4. Bivariate and multivariate association between clinical parameters and the methylation levels of cg25953130. Table S5. Serum lipids, Hcy and FPG levels of mouse. Table S6. Primers for RT-qPCR. Figure S1. Spearman correlation analysis. Figure S2. The effects of Hcy, ox-LDL and FA on DNMT1 and ARID5B expression in primary monocytes. Figure S3. The effect of folic acid on the lipid and Hcy metabolism in mice. Figure S4. Gating strategy for human monocyte subsets. Figure S5. Gating strategy for mouse monocyte subsets. Figure S6. Gating strategy for monocyte subsets sorting and purity identification. Figure S7. Purity identification for CD14+ monocytes. [file 13148_2022_1248_MOESM2_ESM.pdf]

**Supplementary Table1.** Clinical characteristics of the enrolled subjects

| Clinical characteristics | Control (n = 210) | CAD (n = 180) | P value |
|---|---|---|---|
| Age (years) | 58.5 (52.50-69.25) | 62 (55.00-67.00) | 0.4834 |
| Sex, male (%) | 129 (61.40%) | 117 (65.00%) | 0.4660 |
| Diabetes, yes (%) | 17 (8.10%) | 39 (21.67%) | **<0.0001** |
| Hypertension, yes (%) | 53 (25.24%) | 116 (64.44%) | **<0.0001** |
| FPG (mmol/L) | 5.36(5.04-5.77) | 5.75(5.14-6.83) | **<0.0001** |
| TC (mmol/L) | 4.51(4.04-4.85) | 4.15(3.51-4.92) | **0.0198** |
| TG (mmol/L) | 1.08(0.87-1.33) | 1.38(0.98-1.87) | **<0.0001** |
| LDL-C (mmol/L) | 2.66(2.26-3.04) | 2.47(1.91-3.16) | 0.2029 |
| HDL-C (mmol/L) | 1.37(1.21-1.58) | 1.12(0.88-1.38) | **<0.0001** |
| Hcy (μmol/L) | 9.20 (2.93-10.95) | 11.52 (3.26-11.52) | **<0.0001** |
| Leukocytes ($\times 10^9$) | 5.64(4.80-6.41) | 5.95(4.96-7.04) | **0.0383** |
| Neutrophil ($\times 10^9$) | 3.05(2.55-3.60) | 3.72(2.98-4.69) | **<0.0001** |
| Monocyte ($\times 10^9$) | 0.39(0.32-0.47) | 0.48(0.38-0.63) | **<0.0001** |
| Lymphocyte ($\times 10^9$) | 1.92(1.62-2.32) | 1.50(1.16-1.83) | **<0.0001** |

Data are presented as median (interquartile range) or n (%).

Abbreviations: FPG, fasting plasma glucose; TC, total cholesterol; TG, triglyceride; LDL-C, low-density lipoprotein cholesterol; HDL-C, high-density lipoprotein cholesterol; Hcy, homocysteine. Significant P values were in bold.

**Supplementary Table2.** Clinical characteristics of the randomly selected subjects

| Clinical characteristics | Control (n = 112) | CAD (n = 110) | P value |
|---|---|---|---|
| Age (years) | 60 (53.25-70.00) | 61 (55.00-67.00) | 0.8515 |
| Sex, male (%) | 70 (48.95%) | 73 (51.05%) | 0.5477 |
| Diabetes, yes (%) | 5 (4.46%) | 22 (20.00%) | **0.0004** |
| Hypertension, yes (%) | 33 (32.35%) | 69 (67.65%) | **<0.0001** |
| FPG (mmol/L) | 5.43(5.13-5.76) | 5.75(5.15-6.74) | **0.0008** |
| TC (mmol/L) | 4.49(4.02-4.84) | 4.16(3.45-4.94) | 0.1588 |
| TG (mmol/L) | 1.12(0.86-1.36) | 1.49(0.97-1.88) | **<0.0001** |
| LDL-C (mmol/L) | 2.68(2.25-3.04) | 2.48(1.89-3.15) | 0.4028 |
| HDL-C (mmol/L) | 1.41(1.24-1.63) | 1.12(0.88-1.37) | **<0.0001** |
| Hcy (μmol/L) | 8.94 (2.93-8.94) | 11.52 (3.26-11.52) | **<0.0001** |
| Leukocytes ($\times10^9$) | 5.69(4.80-6.13) | 5.80(4.94-6.75) | 0.7891 |
| Neutrophil ($\times10^9$) | 2.99(2.51-3.67) | 3.57(2.72-4.33) | **0.0018** |
| Monocyte ($\times10^9$) | 0.40(0.33-0.48) | 0.44(0.37-0.54) | **0.0041** |
| Lymphocyte ($\times10^9$) | 1.96(1.66-2.33) | 1.55(1.23-1.86) | **<0.0001** |

Data are presented as median (interquartile range) or n (%).
Abbreviations: FPG, fasting plasma glucose; TC, total cholesterol; TG, triglyceride; LDL-C, low-density lipoprotein cholesterol; HDL-C, high-density lipoprotein cholesterol; Hcy, homocysteine. Significant P values were in bold.

**Supplementary Table 3.** Independent risk factors for CAD

| Parameters | Multivariate regressions | |
|---|---|---|
| | β(95%CI) | P |
| FPG | 1.56(1.11 - 2.21) | **0.011** |
| LDL-C | 0.55(0.32 - 0.94) | **0.028** |
| TG | 3.63(1.53 - 8.63) | **0.004** |
| HDL-C | 0.31(0.10 - 0.92) | **0.035** |
| Hcy | 1.14(1.05 - 1.24) | **0.003** |
| cg25953130 Methy. | 1.03(1.01 - 1.05) | **0.012** |

Backfoward multivariate regression analysis was used to analyze the independent risk factors for CAD. Adjust for age, FPG, TC, TG, LDL-C, HDL-C, Hcy and leukocyte count.

Abbreviations: Methy., methylation; FPG, fasting plasma glucose; TC, total cholesterol; TG, triglyceride; LDL-C, low-density lipoprotein cholesterol; HDL-C, high-density lipoprotein cholesterol; Hcy, homocysteine; 95%CI, 95% confidence interval.

Significant P values were in bold.

**Supplementary Table 4.** Bivariate and multivariate association between clinical parameters and the methylation levels of cg25953130

| Groups | Parameters | cg25953130 methylation levels（%） | | | |
|---|---|---|---|---|---|
| | | Univariate correlations | | Multivariate regressions | |
| | | r | P | β(95%CI) | P |
| Control | Age | -0.2305 | **0.0145** | -0.24(-0.59 - -0.10) | **0.0059** |
| | FPG | -0.1795 | 0.0606 | - | - |
| | TC | 0.0736 | 0.4404 | - | - |
| | TG | 0.2804 | **0.0027** | 0.21 (1.06 - 21.25) | **0.0307** |
| | LDL-C | 0.2984 | **0.0014** | - | - |
| | HDL-C | -0.1617 | 0.0884 | -0.03(-13.70 - 9.50) | 0.7206 |
| | Hcy | 0.2589 | **0.0058** | 0.27 (0.45 - 2.04) | **0.0024** |
| | Leukocytes | -0.1062 | 0.2649 | - | - |
| CAD | Age | 0.0986 | 0.3054 | 0.14(-0.09 - 0.66) | 0.1375 |
| | FPG | -0.0504 | 0.6080 | - | - |
| | TC | -0.0781 | 0.4354 | - | - |
| | TG | 0.0869 | 0.3848 | -0.01(-2.78 - 2.59) | 0.9435 |
| | LDL-C | -0.0775 | 0.4389 | - | - |
| | HDL-C | -0.2907 | **0.0030** | -0.33(-23.82 - -6.33) | **0.0009** |
| | Hcy | -0.1118 | 0.2492 | -0.08(-0.73 - 0.29) | 0.3867 |
| | Leukocytes | -0.1000 | 0.2986 | - | - |

In order to control the influence of confounding factors on the linear regression model, in the multivariate regression analysis, we first use Stepwise's statistical method to eliminate the variables with collinearity and establish the optimal regression model, in which the age, TG, Hcy and HDL-C parameters are incorporate into the regression model.

Abbreviations: FPG, fasting plasma glucose; TC, total cholesterol; TG, triglyceride; LDL-C, low-density lipoprotein cholesterol; HDL-C, high-density lipoprotein cholesterol; Hcy, homocysteine; 95%CI, 95% confidence interval.

Significant P values were in bold.

**Supplementary Table 5.** Serum lipids, Hcy and FPG levels of mouse

| groups | Contents | | | | | |
|---|---|---|---|---|---|---|
| | TC (mmol/L) | TG (mmol/L) | LDL-C(mmol/L) | HDL-C(mmol/L) | FPG (mmol/L) | Hcy(μmol/L) |
| G1 | 2.54±0.58 | 0.65±0.20 | 0.24±0.08 | 1.59±0.20 | 3.14±1.21 | 15.04±1.75 |
| G2 | 14.94±2.47 | 0.75±0.08 | 2.22±0.44 | 0.71±0.13 | 3.24±1.13 | 13.97±2.10 |
| G3 | 20.46±7.87 | 0.97±0.60 | 4.09±2.46 | 0.66±0.15 | 5.21±1.79 | 9.21±1.14 |
| G4 | 13.38±2.70 | 0.65±0.22 | 1.86±0.42 | 0.72±0.10 | 3.98±1.06 | 12.83±1.27 |
| G5 | 14.89±2.71 | 0.95±0.29 | 2.45(2.19-2.50) | 0.82±0.16 | 2.78±0.53 | 28.77±7.85 |
| G6 | 11.16±1.91 | 0.66±0.13 | 1.95±0.17 | 0.55±0.11 | 2.57±0.73 | 19.52±3.95 |
| G7 | 15.98±3.49 | 0.77±0.15 | 2.54±0.67 | 0.75±0.15 | 3.82±0.84 | 30.90±8.14 |

Data are presented as mean ± SD (standard deviation) or as median (interquartile range).

Mice were divided into seven subgroups: G1, ApoE-WT + ND; G2, ApoE-/- + ND; G3, ApoE-/- + HFD; G4, ApoE-/- + HFD + FA; G5, ApoE-/- + ND + Hcy; G6, ApoE-/- + ND + Hcy + FA; G7, ApoE-/- + HFD + Hcy + FA.

Abbreviations: FPG, fasting plasma glucose; TC, total cholesterol; TG, triglyceride; LDL-C, low-density lipoprotein cholesterol; HDL-C, high-density lipoprotein cholesterol; Hcy, homocysteine; WT, wild type; ND, normal diet; HFD, high fat diet; FA, folic acid.

**Supplementary Table 6.** Primers for RT-qPCR

| **Species** | **Gene Name** | **Primers（5'to3'）** |
|---|---|---|
| Human | GAPDH | Forward：GAAGGTGAAGGTCGGAGTC |
| | | Reverse：GAAGATGGTGATGGGATTTC |
| | DNMT1 | Forward：ACCGCTTCTACTTCCTCGAGGCCTA |
| | | Reverse：GTTGCAGTCCTCTGTGAACACTGTGG |
| | ARID5B | Forward：GAATTAGGCGGTAATCCTGGGAG |
| | | Reverse：TCCGAGGTTTGATTGGAGGCAG |
| | MCP-1 | Forward：AAGTGTCCCAAAGAAGCTGTG |
| | | Reverse：AGTTTGGGTTTGCTTGTCCAG |
| | CCR2 | Forward：TACGGTGCTCCCTGTCATAAA |
| | | Reverse：TAAGATGAGGACGACCAGCAT |
| | CD86 | Forward：CTGCTCATCTATACACGGTTACC |
| | | Reverse：GGAAACGTCGTACAGTTCTGTG |
| | IL-10 | Forward：GACTTTAAGGGTTACCTGGGTTG |
| | | Reverse：TCACATGCGCCTTGATGTCTG |
| | Arg-1 | Forward：TGGACAGACTAGGAATTGGCA |
| | | Reverse：CCAGTCCGTCAACATCAAAACT |
| | TNF-α | Forward：AGAACTCACTGGGGCCTACA |
| | | Reverse：GCTCCGTGTCTCAAGGAAGT |
| Mouse | GAPDH | Forward：AGGTCGGTGTGAACGGATTTG |
| | | Reverse：TGTAGACCATGTAGTTGAGGTCA |
| | DNMT1 | Forward：AAGAATGGTGTTGTCTACCGAC |
| | | Reverse：CATCCAGGTTGCTCCCCTTG |
| | ARID5B | Forward：TTCCTCCCCGAAGACACTCC |
| | | Reverse：CTGTCCGTTTCTCCCGAAGG |
| | MCP-1 | Forward：TAAAAACCTGGATCGGAACCAAA |
| | | Reverse：GCATTAGCTTCAGATTTACGGGT |
| | TNF-α | Forward：CCCTCACACTCAGATCATCTTCT |
| | | Reverse：GCTACGACGTGGGCTACAG |

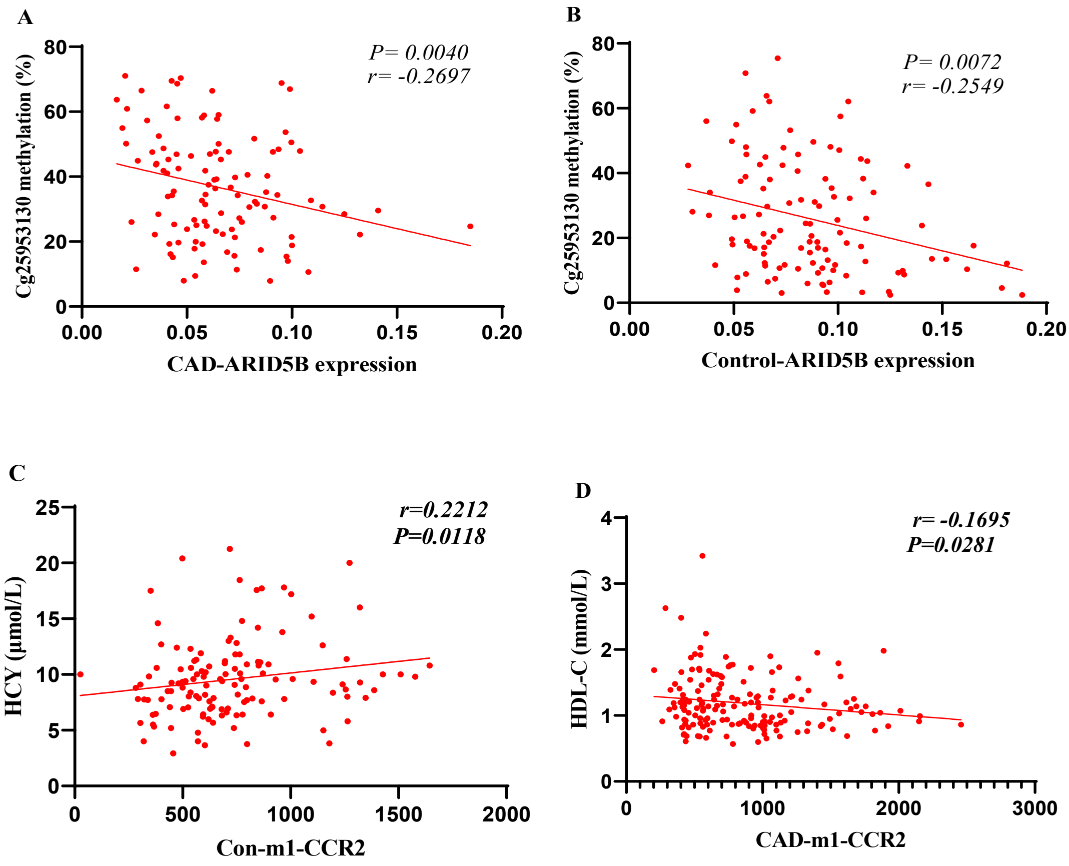


**Supplementary Fig. 1.** Spearman correlation analysis. (A, B) Spearman correlation between the methylation levels of cg25953130 and the expression of ARID5B in the CAD and control groups. (C) Spearman correlation between Hcy levels and the expression of CCR2 on classical monocytes in the control group. (D) Spearman correlation between HDL-C levels and the expression of CCR2 on classical monocytes in the CAD group.

Abbreviations: m1, classical monocytes; Con, control.

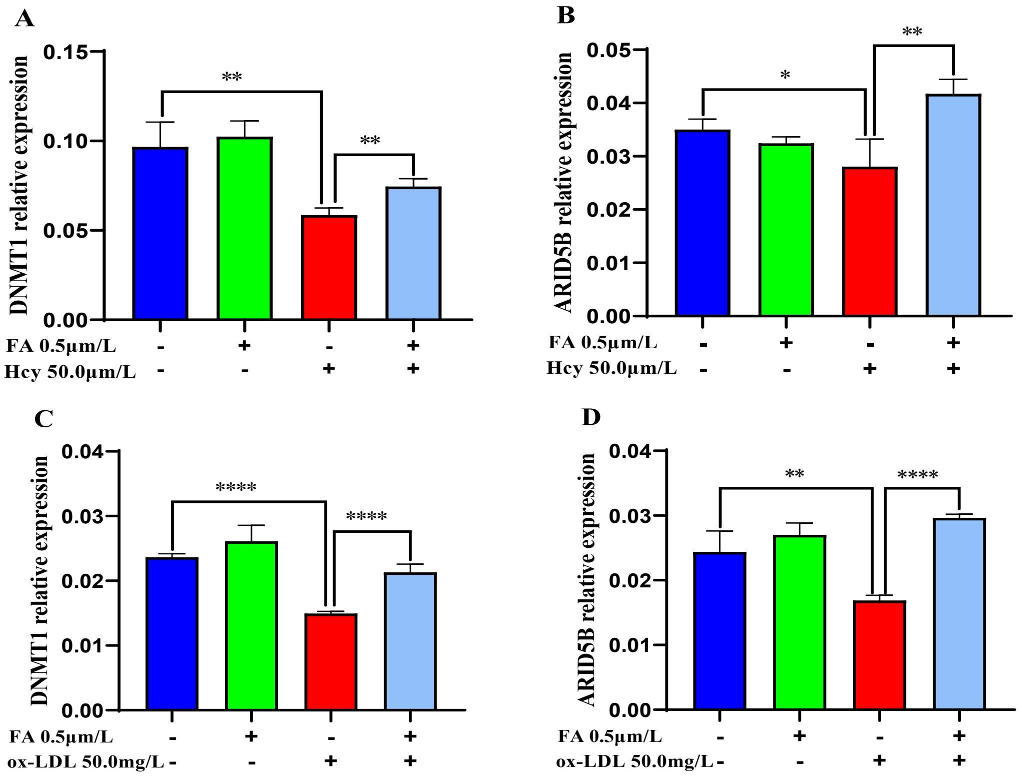


**Supplementary Fig. 2.** The effects of Hcy, ox-LDL and FA on DNMT1 and ARID5B expression in primary monocytes. (A, B) The effect of folic acid on the expression of DNMT1 and ARID5B in Hcy-treated primary monocytes. (C, D) The effect of folic acid on the expression of DNMT1 and ARID5B in ox-LDL-treated primary monocytes. All plotted values are the mean ± SE values of at least 3 independent experiments.

Abbreviations: FA, folic acid; Hcy, homocysteine; ox-LDL, oxidized low density lipoprotein.
* $P < 0.05$, ** $P < 0.01$, **** $P < 0.0001$.

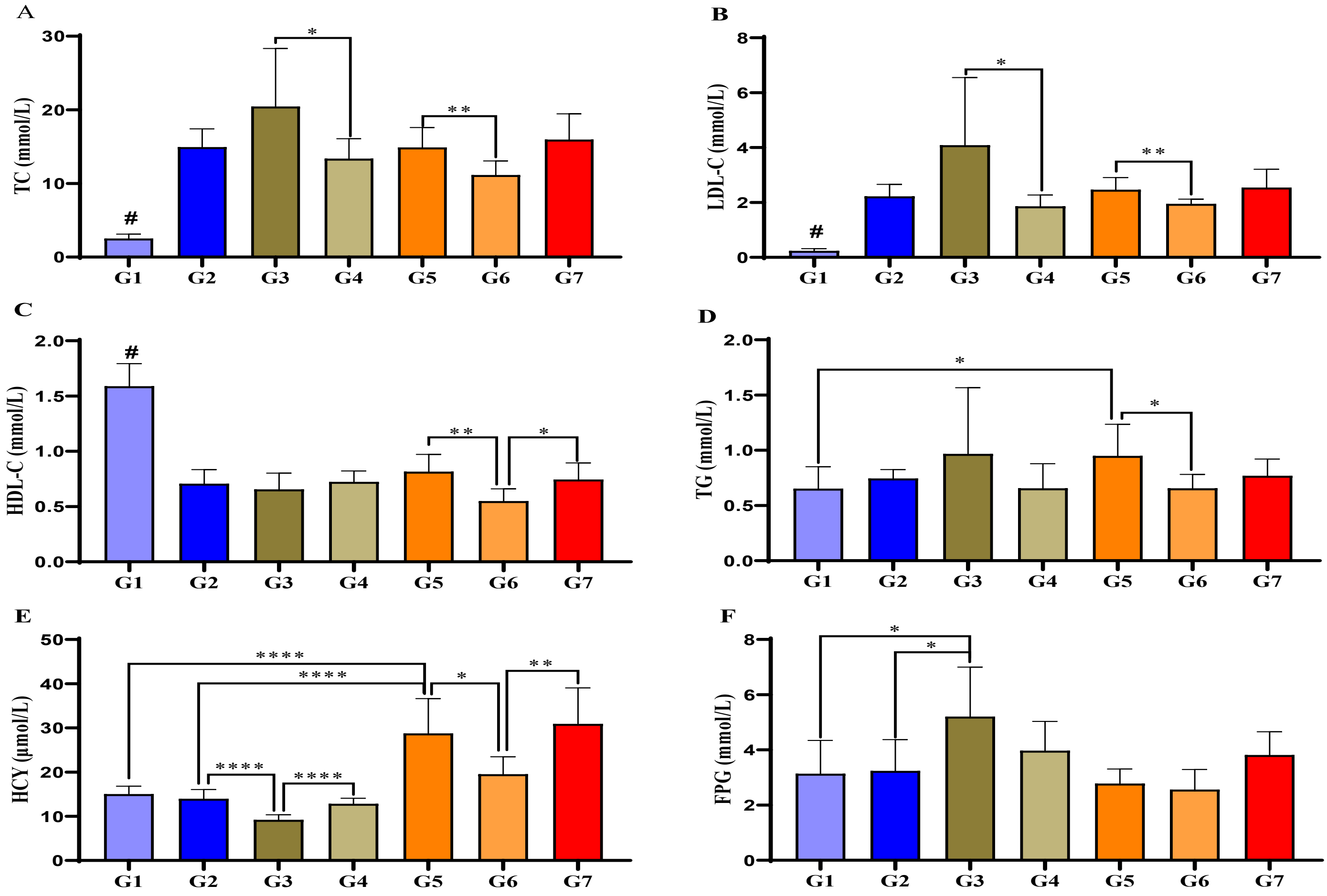


**Supplementary Fig. 3.** The effect of folic acid on the lipid and Hcy metabolism in mice. (A-D) The effect of folic acid on serum TC, LDL-C, HDL-C and TG in mice. (E) The effect of folic acid on serum Hcy in mice. (F) The effect of folic acid on serum FPG in mice. Mice were divided into seven subgroups: G1, ApoE-WT + ND; G2, ApoE-/- + ND; G3, ApoE-/- + HFD; G4, ApoE-/- + HFD + FA; G5, ApoE-/- + ND + Hcy; G6, ApoE-/- + ND + Hcy + FA; G7, ApoE-/- + HFD + Hcy + FA. Abbreviations: FPG, fasting plasma glucose; TC, total cholesterol; TG, triglyceride; LDL-C, low-density lipoprotein cholesterol; HDL-C, high-density lipoprotein cholesterol; Hcy, homocysteine; WT, wild type; ND, normal diet; HFD, high fat diet; FA, folic acid.

#, the G1 group was statistically different from the other 6 groups; * $P < 0.05$, ** $P < 0.01$, *** $P < 0.001$, **** $P < 0.0001$.

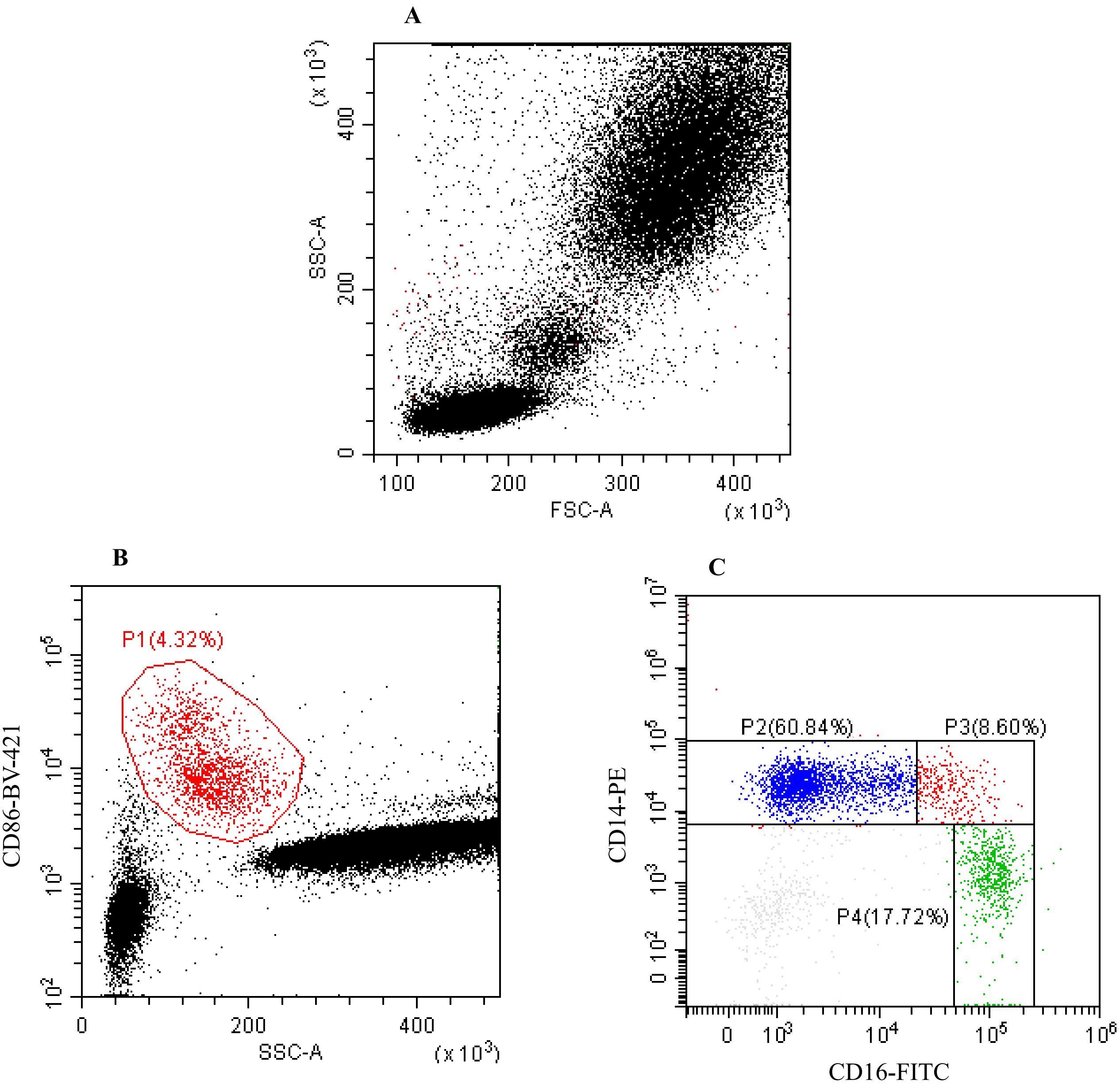


**Supplementary Fig. 4.** Gating strategy for human monocyte subsets. Circulating leukocytes in FSC/SSC dot plot were presented in supplementary Fig. 4A. In CD86/SSC dot plot, CD86 positive monocytes were firstly gated (P2, B). Subsequently, in the CD14/CD16 dot plot (C), based on the expression of CD14 and CD16, the identified monocytes were divided into classical (P2, CD14++CD16-), intermediate (P3, CD14++CD16+) and nonclassical (P4, CD14+CD16++) subsets.

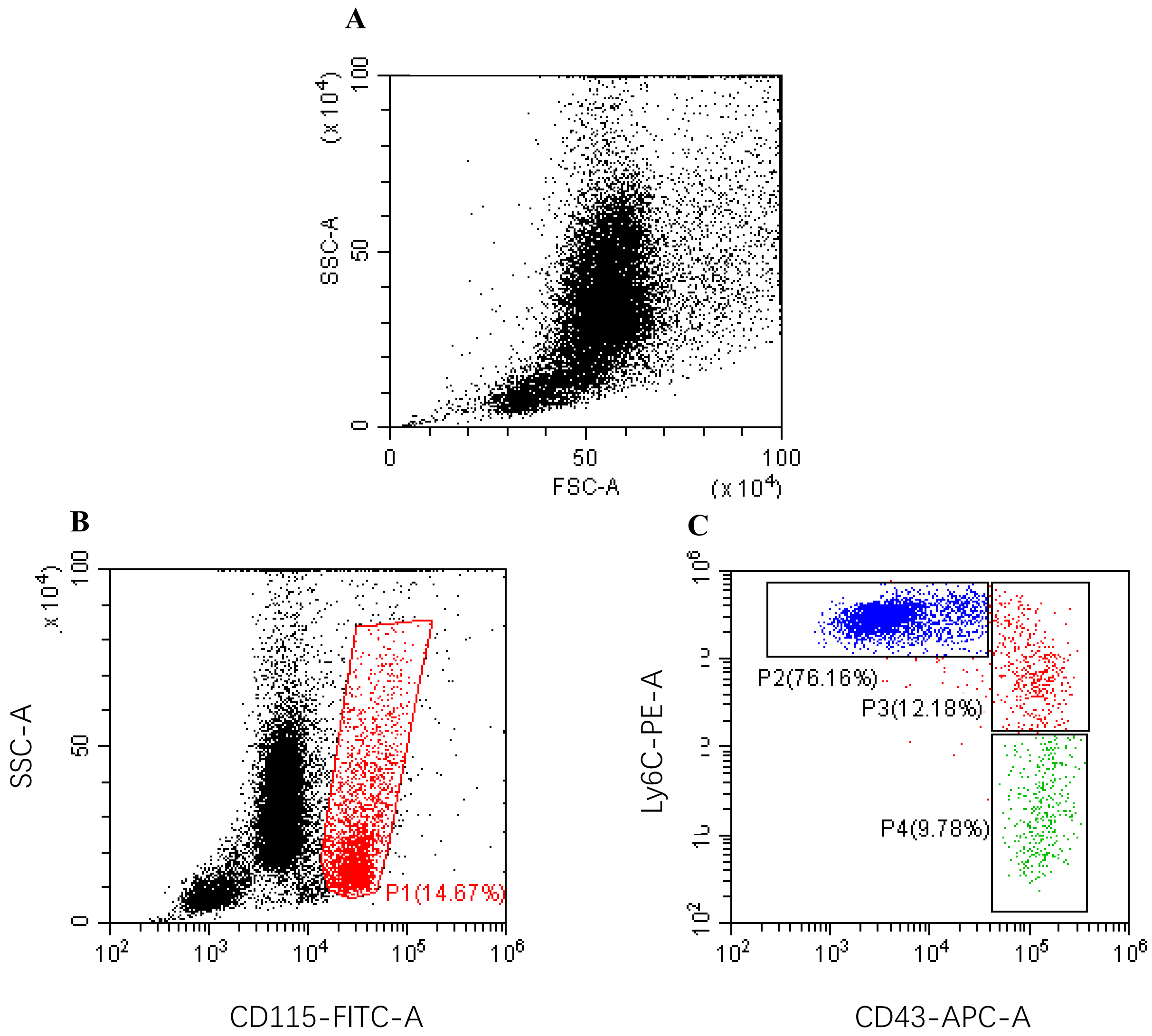


**Supplementary Fig. 5.** Gating strategy for mouse monocyte subsets. Circulating mouse leukocytes in FSC/SSC dot plot were presented in supplementary Fig. 5A. In SSC/CD115 dot plot, CD115 positive monocytes were firstly gated (P1, B). Subsequently, in the Ly6C/CD43 dot plot (C), based on the expression of Ly6C and CD43, the identified monocytes were divided into classical (P2, Ly6C++CD43+), intermediate (P3, Ly6C++CD43++) and nonclassical (P4, Ly6C+CD43++) subsets.

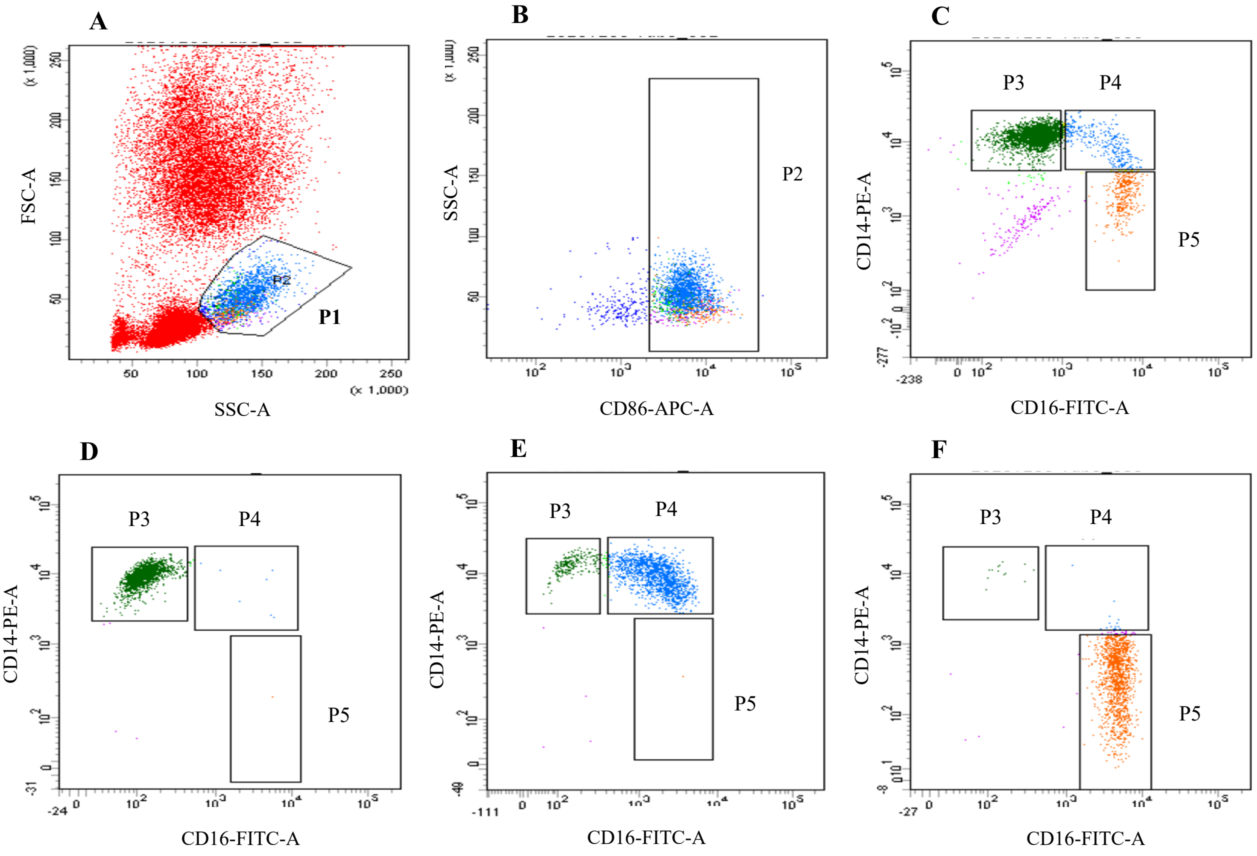


**Supplementary Fig. 6.** Gating strategy for monocyte subsets sorting and purity identification. Circulating monocytes in FSC/SSC dot plot were presented in supplementary Fig. 6A (P1). In CD86/SSC dot plot, CD86 positive monocytes were firstly gated (P2, B). Subsequently, in the CD14/CD16 dot plot, based on the expression of CD14 and CD16 (C), the identified monocytes were divided into classical (P3, CD14++CD16-), intermediate (P4, CD14++CD16+) and nonclassical (P5, CD14+CD16++) subsets. The monocyte subsets obtained by FCM sorting had good purity (D-F).

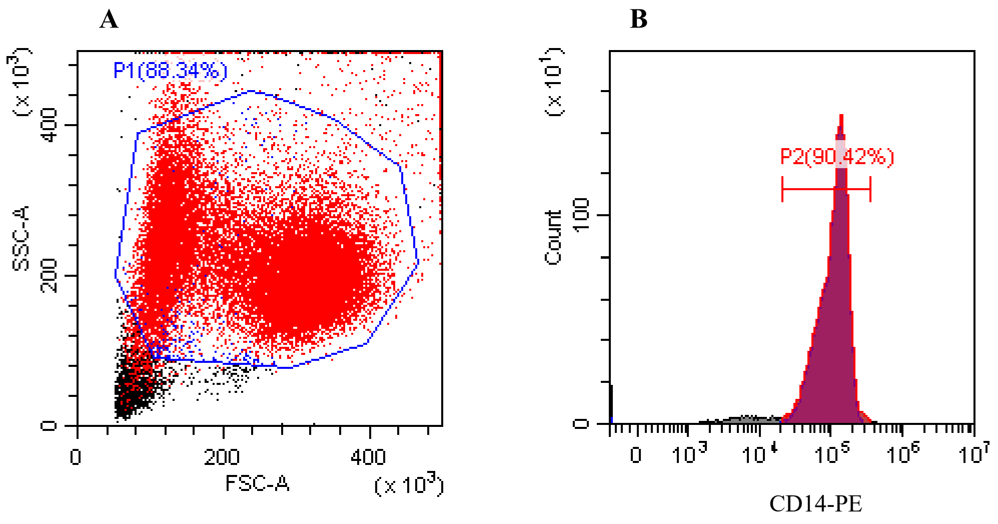


**Supplementary Fig. 7.** Purity identification for CD14+ monocytes. Flow cytometry was used to identify the purity of the obtained monocytes labeled with CD14 monoclonal fluorochrome-conjugated antibody. (A) Gating strategy for monocyte CD14+ monocytes. (B) Purity of the CD14+ monocytes.
